# Alpinetin Exhibits Antioxidant and Anti-Inflammatory Effects in C57BL/6 Mice with Alcoholic Liver Disease Induced by the Lieber–DeCarli Ethanol Liquid Diet

**DOI:** 10.3390/ijms26010086

**Published:** 2024-12-26

**Authors:** Tatjana Radosavljevic, Milica Brankovic, Jasmina Djuretić, Jelica Grujic-Milanovic, Marijana Kovacic, Jovan Jevtic, Sanja Stankovic, Janko Samardzic, Danijela Vucevic, Vladimir Jakovljevic

**Affiliations:** 1 Institute of Pathophysiology “Ljubodrag Buba Mihailović”, Faculty of Medicine, University of Belgrade, 11000 Belgrade, Serbia; danijela.vucevic@med.bg.ac.rs; 2 Institute of Pharmacology, Clinical Pharmacology and Toxicology, Faculty of Medicine, University of Belgrade, 11000 Belgrade, Serbia; milicabrankovic137@yahoo.com (M.B.); jankomedico@yahoo.es (J.S.); 3 Department of Pathobiology, Faculty of Pharmacy, University of Belgrade, 11000 Belgrade, Serbia; jasmina.djuretic@pharmacy.bg.ac.rs; 4 Institute for Medical Research, National Institute of the Republic of Serbia, Department of Cardiovascular Research, University of Belgrade, 11000 Belgrade, Serbia; jeca@imi.bg.ac.rs; 5 Group of Immunology, Institute for Medical Research, National Institute of the Republic of Serbia, University of Belgrade, 11000 Belgrade, Serbia; marijana.buac@imi.bg.ac.rs; 6 Institute of Pathology ‘Dr Đorđe Joannović’, Faculty of Medicine, University of Belgrade, 11000 Belgrade, Serbia; lordstark90@gmail.com; 7 Centre for Medical Biochemistry, University Clinical Centre of Serbia, 11000 Belgrade, Serbia; sanjast2013@gmail.com; 8 Department of Biochemistry, Faculty of Medical Sciences, University of Kragujevac, 34000 Kragujevac, Serbia; 9 Department of Physiology, Faculty of Medical Sciences, University of Kragujevac, 34000 Kragujevac, Serbia; drvladakgbg@gmail.com; 10 Center of Excellence for the Study of Redox Balance in Cardiovascular and Metabolic Disorders, University of Kragujevac, 34000 Kragujevac, Serbia; 11 Department of Human Pathology, First Moscow State Medical University I.M. Sechenov, Trubetskaya Street 8, Str. 2, 119991 Moscow, Russia

**Keywords:** alpinetin, alcohol-associated liver disease, oxidative/nitrosative stress, ER stress

## Abstract

Alcohol-associated liver disease (ALD) is a common non-communicable chronic liver disease characterized by a spectrum of conditions ranging from steatosis and alcohol-associated steatohepatitis (AH) to fibrosis, cirrhosis, and hepatocellular carcinoma (HCC). The pathogenesis of ALD involves a complex interplay of various molecular, biochemical, genetic, epigenetic, and environmental factors. While the mechanisms are well studied, therapeutic options remain limited. Alpinetin, a natural flavonoid with antioxidant and anti-inflammatory properties, has shown potential hepatoprotective effects, though its efficacy in ALD remains unexplored. This study investigated the hepatoprotective effects of alpinetin using a Lieber–DeCarli ethanol liquid diet model of ALD in C57BL/6 mice. Mice were divided into three groups: the control group, the ethanol group, and the ethanol group treated with alpinetin. Serum activity of ALT, AST, γ-GT, and ALP was measured to assess liver function, along with antioxidative and oxidative/nitrosative stress markers in liver tissue. Pro-inflammatory cytokines and endoplasmic reticulum (ER) stress parameters in liver tissue were also evaluated. Histological assessment of disease activity was performed using the SALVE grading and staging system. Treatment with alpinetin significantly reduced serum levels of ALT, AST, γ-GT, and oxidative/nitrosative stress markers while increasing antioxidative markers. The levels of pro-inflammatory cytokines and ER stress parameters were significantly decreased. Histological analysis demonstrated reduced steatosis, hepatocyte ballooning, and inflammation. These findings suggest that alpinetin holds promise as a potential therapeutic agent for managing ALD.

## 1. Introduction

Alcohol-associated liver disease (ALD) is one of the most common non-communicable chronic liver diseases worldwide [1]. It represents a histological spectrum of diseases, ranging from alcohol-associated fatty liver (simple steatosis) and alcohol-associated steatohepatitis (AH) to fibrosis, cirrhosis, and hepatocellular carcinoma (HCC) [2]. ALD develops in over 90% of individuals who consume more than 60 g of alcohol per day, while about 35% of patients with ALD progress to alcohol-associated steatohepatitis (ASH) [3]. Furthermore, 8% to 20% of individuals who chronically consume alcohol develop cirrhosis, and approximately 2% of these cases advance to HCC [4]. The pathogenesis of ALD involves a complex interplay of various molecular, biochemical, genetic, epigenetic, and environmental factors that are not fully understood [5]. However, several key factors and processes are known to contribute to the development and progression of ALD. Alcohol is primarily metabolized in the liver by enzymes such as alcohol dehydrogenase (ADH) and cytochrome P450 2E1 (CYP2E1). This metabolism produces acetaldehyde, a toxic compound that can cause cellular and DNA damage, leading to oxidative stress and activation of inflammatory pathways [6]. Moreover, alcohol metabolism generates reactive oxygen species (ROS), which can induce lipid peroxidation, protein oxidation, and DNA damage in liver cells. ROS production impairs mitochondrial function, leading to ATP depletion and further oxidative stress [7]. Oxidative stress also disrupts endoplasmic reticulum (ER) function, causing ER stress and contributing to liver cell injury and apoptosis [8,9,10]. Chronic alcohol consumption activates the immune system, initiating liver inflammation. Kupffer cells, specialized liver macrophages, play a key role in mediating inflammation by producing pro-inflammatory cytokines such as tumor necrosis factor-alpha (TNF-α) and interleukin-1 (IL-1) [6,11]. Moreover, alcohol consumption can compromise the gut barrier, allowing bacterial toxins such as lipopolysaccharides to enter the bloodstream and reach the liver. These toxins trigger an immune response within the liver, exacerbating inflammation and causing liver damage [6]. Chronic inflammation and injury prompt the activation of hepatic stellate cells (HSCs), which produce collagen, profibrogenic mediators, and other extracellular matrix components, resulting in fibrosis. Continued liver injury and fibrosis can progress to cirrhosis, a condition that significantly impairs liver function and may eventually lead to HCC [6,11]. Genetic mutations, epigenetic changes (including DNA methylation, histone modifications, and mRNA regulation), and environmental factors can all affect an individual’s susceptibility to the development and progression of ALD [5]. While we have gained valuable insights into the pathogenesis of ALD, further research is necessary to fully elucidate the precise triggers for disease progression and to identify potential therapeutic targets.

The pathogenesis of ALD has been extensively studied in various experimental murine models, including the intragastric feeding model (Tsukamoto–French model) [12], Lieber–DeCarli diet-induced model [13], acute gavage [14], and *ad libitum* ethanol intake in drinking water [15]. A more recent addition to these models is the chronic and binge ethanol feeding model developed by the National Institute on Alcohol Abuse and Alcoholism (NIAAA model) [16]. Chronic binge ethanol feeding causes significant elevation in serum alanine aminotransferase levels and hepatic steatosis, accompanied by inflammation, without obvious fibrosis [16,17]. Currently, the most widely used model for studying alcoholic liver injury is the Lieber–DeCarli diet-induced model [18]. This model involves feeding rodents with ethanol-containing liquid diets over an extended period, mimicking chronic alcohol consumption seen in humans. It allows studying various aspects of ALD, including steatosis, inflammation, fibrosis, and cirrhosis [17,18].

Alpinetin, a natural flavonoid found in *Alpinia katsumadai Hayata*, shows promise as a hepatoprotective agent [19,20,21,22,23,24]. In an experimental model of NAFLD, alpinetin exhibits strong antioxidant activity, scavenging free radicals and reducing oxidative stress in hepatocytes [21]. Alpinetin has been shown to suppress inflammatory pathways in the liver by inhibiting the production of pro-inflammatory cytokines and reducing the activation of inflammatory cells [21]. Research has indicated that alpinetin may inhibit the activation of hepatic stellate cells, which are responsible for the production of collagen and other extracellular matrix proteins involved in liver fibrosis [20]. Alpinetin has also been reported to modulate lipid metabolism in the liver, promoting the clearance of fatty acids and preventing the accumulation of triglycerides [21,22]. However, there is currently no accessible information on the hepatoprotective activity of alpinetin in ALD.

## 2. Results

### 2.1. The Effects of Alpinetin on Body Weight, Liver Weight, and Liver Index

The Lieber–DeCarli ethanol diet resulted in a significant decrease in body weight (*p* < 0.001) and a significant increase in liver weight (*p* < 0.001) in the mice. Consequently, the liver index (the liver/body weight ratio) was significantly higher in the E group compared to the control mice (*p* < 0.001). Although the liver index in the E+Alp group was higher compared to control values (*p* < 0.05), it was significantly lower (*p* < 0.01) compared to the E group. As expected, the body weight loss in the alpinetin-treated group was significantly reduced in comparison to the E group (*p* < 0.001) (Table 1, Figure S1).

### 2.2. The Effects of Alpinetin on Serum ALT, AST, γ-G, and ALP Activity

The serum activity of ALT and AST was significantly increased in the E group compared to the control group (*p* < 0.001). On the other hand, the activity of these enzymes decreased significantly in the E+Alp group compared to the E group (*p* < 0.001). Similarly, γ-GT activity was significantly lower in the E+Alp group in comparison to the E group (*p* < 0.001) (Table 2). There was no statistically significant difference in serum ALP activity in all investigated groups (*p* > 0.05).

### 2.3. The Effects of Alpinetin on Oxidative/Nitrosative Parameters

**Figure 1:**
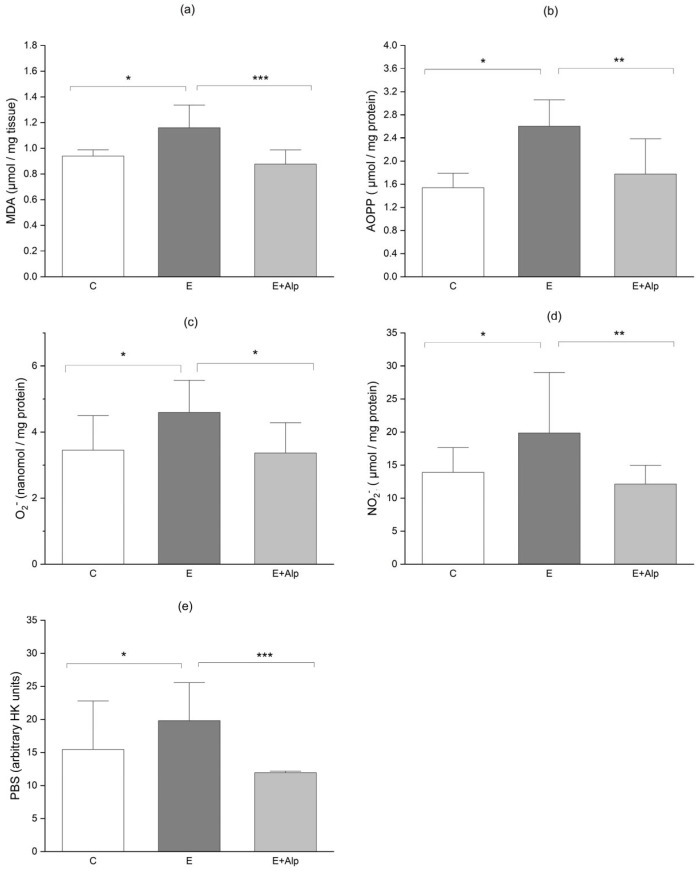
The effects of alpinetin on oxidative/nitrosative stress parameters: (**a**) MDA, (**b**) AOPP, (**c**) O_2_^−^, (**d**) NO_2_^−^, (**e**) PAB. The values are represented by mean ± SD; *** *p* < 0.001, ** *p* < 0.01, * *p* < 0.05. Abbreviations: MDA—malondialdehyde; AOPP—advanced oxidation protein product; (**c**) O_2_^−^ superoxide anion radical; (**d**) NO_2_—nitrite; (**e**) PAB—pro-oxidant–antioxidant balance; C—control group; E—ethanol group; E+Alp—ethanol + alpinetin group.

The concentration of malondialdehyde (MDA) in liver tissue, a marker of lipid peroxidation, was significantly increased in the E group (1.16 ± 0.14 μmol/g tissue) compared to control values (0.94 ± 0.04 μmol/g tissue) (*p* < 0.05). In contrast, hepatic MDA concentration was significantly lower in the E+Alp group (0.89 ± 0.08 μmol/g tissue) in comparison with the E group (*p* < 0.001) (Figure 1a).

The advanced oxidation protein products (AOPPs) level in liver tissue was significantly elevated in the E group (2.60 ± 0.39 μmol/mg protein) compared to the control group (1.79 ± 0.76 μmol/mg protein) (*p* < 0.05). Conversely, the E+Alp group showed a significant reduction in liver AOPP levels (1.77 ± 0.38 μmol/mg protein) compared to the E group (*p* < 0.01) (Figure 1b).

Liver superoxide anion radical (O_2_^−^) concentration was significantly increased in the E group (4.59 ± 0.74 nmol/mg protein) in comparison to control values (3.27 ± 0.59 nmol/mg protein) (*p* < 0.05). In contrast, liver O_2_^−^ level was significantly decreased in the E+Alp group (3.63 ± 1.29 nmol/mg protein) compared to the E group (*p* < 0.05) (Figure 1c).

The concentration of nitrite (NO_2_^−^ in the E group was significantly elevated (19.86 ± 5.81 μmol/mg protein) compared to the control group (13.89 ± 3.70 μmol/mg protein) (*p* < 0.05). However, alpinetin supplementation significantly reduced liver NO_2_^−^ levels (12.13 ± 4.79 μmol/mg protein) in comparison to the E group (*p* < 0.01) (Figure 1d).

The pro-oxidant–antioxidant balance (PAB) was significantly increased in the E group (19.81 ± 3.89 arbitrary HK units) compared to control values (15.44 ± 4.39 arbitrary HK units) (*p* < 0.05). In contrast, the E+Alp group demonstrated a significant reduction in PAB levels (11.94 ± 0.53 arbitrary HK units) compared to the E group (*p* < 0.01) (Figure 1b).

### 2.4. The Effects of Alpinetin on Antioxidative Parameters

**Figure 2:**
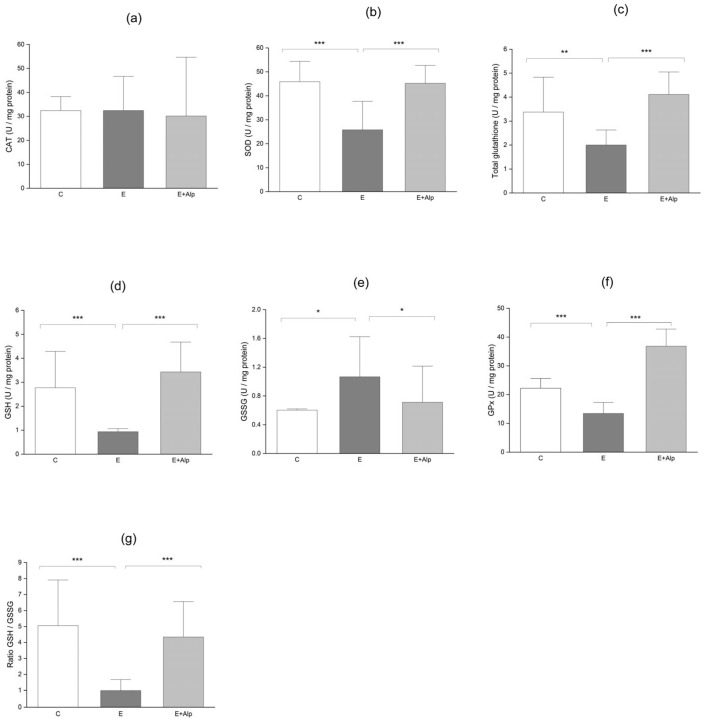
The effects of alpinetin on antioxidative parameters: (**a**) CAT, (**b**) SOD, (**c**) total glutathione, (**d**) GSH, (**e**) GSSG, (**f**) GPx, (**g**) GSH/GSSG ratio. The values are represented by mean ± SD; *** *p* < 0.001, ** *p* < 0.01, * *p* < 0.05, respectively. Abbreviations: CAT—catalase; SOD—superoxide dismutase; GSH—reduced glutathione; GSSG—oxidized glutathione; GPx—glutathione peroxidase; C—control group; E—ethanol group; E+Alp—ethanol + alpinetin group.

CAT activity in the liver did not exhibit significant differences between the E and E+Alp groups compared to the control group. Additionally, treatment with alpinetin did not significantly affect CAT activity in the E+Alp group compared to the E group (32.42 ± 11.33, 30.11 ± 18.66, and 32.38 ± 18.84 U/mg protein for the E, E+Alp, and control group, respectively) (Figure 2a).

Total liver SOD activity was significantly reduced in the E group (25.81 ± 7.73 U/mg protein) compared to control values (45.89 ± 9.03 U/mg protein) (*p* < 0.001). However, alpinetin treatment significantly increased SOD activity in the E+Alp group (45.21 ± 10.93 U/mg protein) compared to the E group (*p* < 0.001) (Figure 2b).

Further analysis of hepatic antioxidant capacity demonstrated a significant decrease in reduced GSH, total glutathione (GSH), glutathione peroxidase (GPx), and increased oxidized glutathione (GSSH) levels in the E group compared to control values (reduced GSH, total GSH, GPx, GSSH; *p* < 0.001, *p* < 0.01, and *p* < 0.05, respectively) (Figure 2c–f). Further, the GSH/GSSH ratio, a marker of oxidative stress, was significantly reduced in the E group compared to the control group (*p* < 0.001). Alpinetin supplementation led to a significant increase in total GSH reduced GSH, GPx, and the GSH/GSSH ratio, while significantly decreasing oxidized GSH levels in comparison to the E group (*p* < 0.001 and *p* < 0.05, respectively) (Figure 2c–g).

### 2.5. The Effects of Alpinetin on Cytokines (IFN-γ and IL-4) and MPO Activity

**Figure 3:**
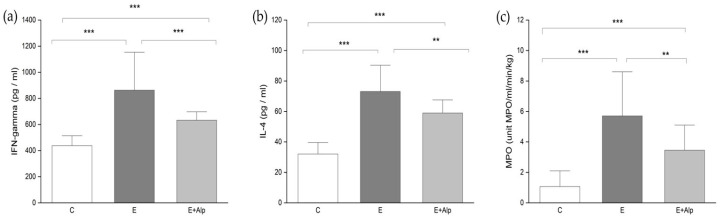
The effects of alpinetin on (**a**) IFN-γ, (**b**) IL-4, and (**c**) MPO activity. The values are represented by mean ± SD; *** *p* < 0.001, ** *p* < 0.01. Abbreviations: IFN-γ—interferon-gamma; IL-4—interleukin 4; MPO—myeloperoxidase; C—control group; E—ethanol group; E+Alp—ethanol + alpinetin group.

The concentrations of IFN-γ and IL-4 in the liver were significantly elevated in both the E and E+Alp groups compared to control values (*p* < 0.001). However, alpinetin supplementation significantly reduced the liver concentrations of these cytokines in comparison to the E group (*p* < 0.001 and *p* < 0.01, respectively) (Figure 3a,b).

Liver MPO activity was significantly increased in the E and E+Alp groups compared to the control group (*p* < 0.001). In contrast, treatment with alpinetin induced a significant decrease in MPO activity compared to the E group (*p* < 0.01) (Figure 3c).

### 2.6. The Effects of Alpinetin on Endoplasmic Reticulum (ER) Stress Parameters

**Figure 4:**
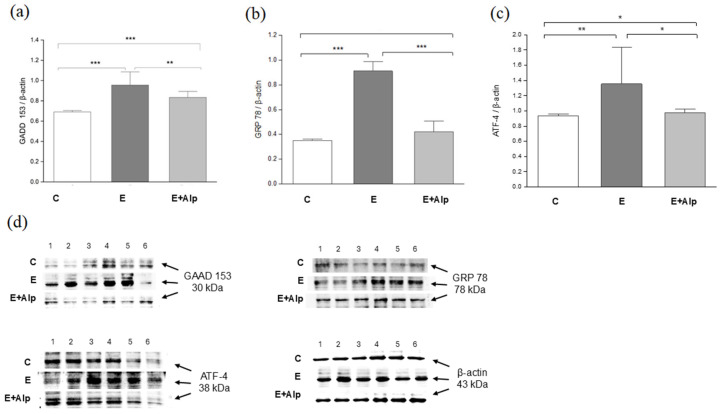
The effects of alpinetin on the expression of the ER stress proteins (**a**) GADD153, (**b**) GRP 78, (**c**) ATF-4, (**d**) Western blot analyses of GADD153, GRP 78, ATF-4, and β-actin in liver tissue (n = 6) (Figure S2). Abbreviations: GADD—growth arrest and DNA damage-inducible gene 153; GRP 78—glucose-regulated protein 78; ATF 4—activating transcription factor 4; C—control group; E—ethanol group; E+Alp—ethanol + alpinetin group. The values are represented by mean ± SD; *** *p* < 0.001, ** *p* < 0.01, * *p* < 0.05.

CADD153 levels in liver tissue were significantly increased in the E group compared to control values (*p* < 0.001). In contrast, alpinetin supplementation resulted in a significant reduction in liver CADD153 levels compared to the E group (*p* < 0.01) (Figure 4a).

CRP78 concentration in liver tissue was significantly elevated in the E group compared to the control group (*p* < 0.001). However, alpinetin supplementation significantly decreased liver CRP78 levels in comparison to the E group (*p* < 0.001) (Figure 4b).

Liver ATF4 concentration was significantly increased in the E group compared to control values (*p* < 0.01). Similarly, alpinetin treatment significantly reduced liver ATF4 levels in comparison to the E group (*p* < 0.05). (Figure 4c)

Hepatic levels of CADD153 and ATF4 in the E+Alp group were significantly increased in comparison to control values (*p* < 0.001, *p* < 0.05, respectively) (Figure 4a–c).

### 2.7. The Effects of Alpinetin on Liver Morphology

The liver of the control mice exhibited normal histological features, with smooth surfaces and uniform hepatocytes, indicating no signs of hepatocellular injury or fatty changes. In contrast, the E group demonstrated significant pathological changes. Numerous hepatocytes exhibited ballooning degeneration, with cytoplasmic clearing. In some areas, hepatocytes also showed the presence of Mallory bodies. Additionally, pronounced steatosis was observed, with many hepatocytes displaying fat vacuoles within the cytoplasm. Neutrophilic infiltration was prominent, as evidenced by the presence of numerous lobular neutrophils between hepatocytes.

**Figure 5:**
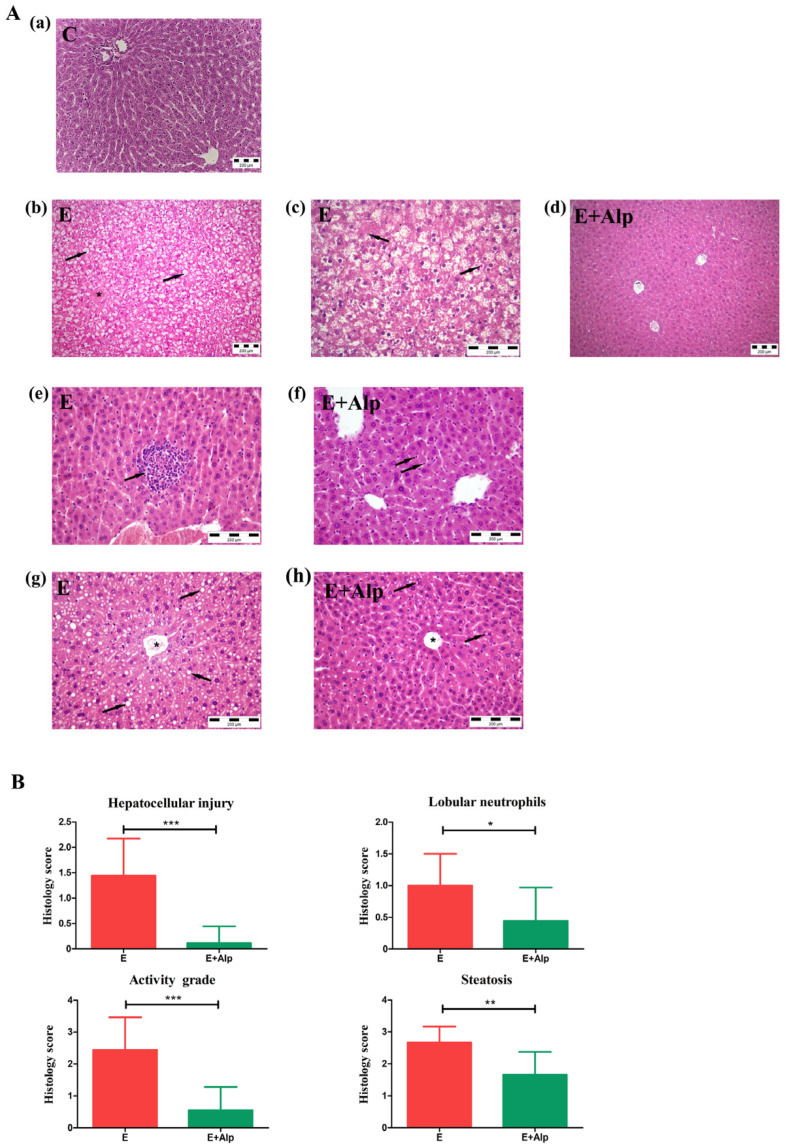
(**A**) Normal liver histology (**A**(a)); liver histology of mice in the E group (**A**(b,c,e,g)); liver histology of mice in the E+Alp group (**A**(d,f,h)). (**A**(b)) hepatocellular injury (ballooning). Numerous hepatocytes are ballooned, showing cytoplasmic clearing (arrows). The image also shows a central vein (asterisk). H&E stain, magnification ×200; (A(c)) Numerous hepatocytes are ballooned, showing cytoplasmic clearing, with some exhibiting Mallory bodies (arrows). H&E stain, magnification ×400; (**A**(g)) Steatosis. Numerous hepatocytes exhibit fatty changes, with fat vacuoles observed in the cytoplasm (arrows). The image also shows a central vein (asterisk). H&E stain, magnification ×400; (**A**(e)) lobular neutrophils. Numerous neutrophils are present between hepatocytes (arrow). H&E stain, magnification ×400. (**A**(d)) The depicted hepatocytes do not show ballooning. H&E stain, magnification ×200; (**A**(h)) A few hepatocytes exhibit fatty changes with sparse microdroplets (arrows). H&E stain, magnification ×400; (**A**(f)) Scattered neutrophils are present between hepatocytes. H&E stain, magnification ×400. (**B**) Histology score of hepatocellular injury, lobular neutrophils (activity grade), and steatosis in the E and E+Alp groups (*** *p* < 0.001, ** *p* < 0.01, * *p* < 0.05 vs. E group).

In the E+Alp group, hepatocytes showed minimal signs of ballooning, and the fatty changes were significantly reduced, with only a small number of hepatocytes displaying sparse fat droplets compared to the E group (*p* < 0.001, *p* < 0.01, respectively). Neutrophil infiltration in the E+Alp group was also less pronounced, with scattered neutrophils observed between hepatocytes in comparison to the E group (*p* < 0.05). The histology score of hepatocellular injury and lobular neutrophils (activity grade) in the E+Alp group was significantly lower compared to the E group (*p* < 0.001). These findings are presented in Figure 5.

## 3. Discussion

In our study using a mouse model of ALD, we observed that alpinetin exhibited a protective effect against liver injury induced by the Lieber–DeCarli ethanol diet. This experimental model, also known as the NIAAA model, was applied in C57BL/6 mice and effectively induced alcoholic steatohepatitis, a condition marked by both micro- and macrovesicular steatosis, inflammatory cell infiltration, hepatocyte ballooning, and the formation of Mallory–Denk bodies [16,25]. As expected, the livers of mice subjected to the Lieber–DeCarli ethanol diet displayed significant morphological changes (Figure 5A(b,c,e,g)). Substantial neutrophil infiltration in the liver tissue was observed, indicating an ongoing inflammatory response. Alpinetin treatment appeared to mitigate some of the severe pathological features associated with ALD. Notably, hepatocyte ballooning, a severe form of liver injury, was not observed in the livers of alpinetin-treated mice (Figure 5A(d)). Furthermore, alpinetin reduced hepatic steatosis and inflammation, as evidenced by the presence of fewer fatty changes with sparse microdroplets and a reduced number of scattered neutrophils in the liver tissue compared to the ethanol-fed control group (Figure 5A(f,h)). These findings suggest that alpinetin may offer a promising strategy to slow the progression of alcoholic steatohepatitis, potentially contributing to the development of more effective treatments for liver diseases associated with alcohol consumption. Studies investigating the effects of alpinetin in non-alcoholic fatty liver disease (NAFLD) and thioacetamide (TAA)-induced liver injury have demonstrated comparable morphological changes to those observed in the present study. These include a significant reduction in fat accumulation, decreased liver cell damage, and less pronounced inflammation [20,21]. Similar to our results, alpinetin consistently mitigated steatosis and hepatic injury in various experimental models, suggesting its potential hepatoprotective role across different liver conditions.

The Lieber–DeCarli ethanol diet led to a significant reduction in body weight in the mice. This weight loss is consistent with chronic alcohol consumption, which is known to cause malnutrition and muscle wasting. Additionally, there was a significant increase in liver weight and liver index (liver/body weight ratio), aligning with the pathophysiology of ALD, where alcohol consumption leads to hepatic steatosis and hepatomegaly due to fat accumulation and inflammation. Our results showed a significant increase in the liver index in mice fed the Lieber–DeCarli ethanol diet compared to the control group (Table 1). This finding aligns with the well-established hepatotoxic effects of alcohol [26,27,28]. Alcohol acts as a direct hepatotoxin, initiating a cascade of metabolic responses that contribute to liver damage [29]. The reduction in body weight and the increase in liver weight can be strongly attributed to the toxic effects of acetaldehyde, a key metabolite in alcohol metabolism. Acetaldehyde is highly reactive and forms protein–aldehyde adducts, which disrupt cellular functions and contribute to liver injury [30]. These adducts impair protein function and promote inflammation and fibrosis, leading to hepatomegaly, as reflected by the increased liver index. The formation of protein–aldehyde adducts and the oxidative stress associated with alcohol metabolism can lead to the release of pro-inflammatory cytokines, which further exacerbate liver inflammation and injury. Chronic inflammation, driven by these immune responses, plays a crucial role in the progression of liver disease [31]. As previously mentioned, chronic alcohol consumption is associated with impaired gut permeability, primarily due to nitro-oxidative stress [32]. Key mechanisms contributing to nitro-oxidative stress include the upregulation of inducible nitric oxide synthase (iNOS), activation of the NF-κB signaling pathway, and increased expression of microRNA-122 (miRNA-122) in intestinal cells [33]. A weakened gut barrier enables the translocation of gut-derived endotoxins into the liver, triggering an immune response that exacerbates liver inflammation and damage [32]. The combination of impaired nutrient absorption and ongoing liver injury creates a vicious cycle that perpetuates malnutrition and liver disease. Furthermore, the increased accumulation of triglycerides, inflammatory cell infiltration, hepatocyte ballooning, and the formation of Mallory–Denk bodies contribute to the elevated liver index observed in mice fed the Lieber–DeCarli ethanol diet. However, the liver index and the percentage of body weight loss were significantly lower in the mice treated with alpinetin compared to the untreated ethanol group (Table 1), indicating the hepatoprotective effects of alpinetin. Additionally, studies have shown that alpinetin may enhance intestinal barrier function by upregulating the expression of tight junction proteins such as ZO-1 and occludin [34,35].

The present study also evaluated the effects of alpinetin on serum levels of the liver enzymes ALT, AST, γ-GT, and alkaline phosphatase (ALP), as well as the AST/ALT ratio, in an experimental model of ALD induced by the Lieber–DeCarli ethanol diet. These enzymes serve as key biomarkers for liver injury, with elevated levels indicating hepatocellular damage and impaired liver function [36]. In the untreated ethanol-fed group, we observed a significant elevation in the serum activity of ALT, AST, and γ-GT compared to the control, reflecting substantial liver injury induced by chronic alcohol consumption (Table 2). This elevation is consistent with the pathophysiology of ALD, where alcohol metabolism leads to hepatocyte injury, inflammation, and cell death [6,11]. Mildly elevated AST activity (ranging between >50 and <400 IU/L) and an AST/ALT ratio greater than 1 are considered as indicators of ALD. Moreover, an AST/ALT ratio exceeding 2 is a marker for AH [37,38]. Both AST and ALT depend on vitamin B6 (pyridoxal-5′-phosphate), and chronic alcohol consumption is commonly associated with a deficiency in this vitamin. Since ALT is more dependent on vitamin B6 for its function than AST, a deficiency in this cofactor leads to reduced ALT activity, further elevating the AST/ALT ratio [39]. Treatment with alpinetin significantly reduced the serum activities of ALT and AST compared to the untreated ethanol group, suggesting a protective effect of alpinetin against alcohol-induced hepatocellular damage (Table 2). This reduction in enzyme activity indicates that alpinetin may help maintain liver integrity by mitigating the extent of hepatocyte injury. The decrease in ALT and AST levels in the alpinetin-treated group is particularly noteworthy, as these enzymes are direct indicators of liver cell health and function [36]. However, the serum activity of ALP was not significantly changed. Similarly to our results, numerous studies have demonstrated that alpinetin reduces the activity of ALT, AST, and γ-GT and mitigates histopathological changes in various experimental models of liver disease. These include galactosamine-induced liver injury [40], TAA and carbon tetrachloride (CCl_4_)-induced liver fibrosis [20], ischemia–reperfusion-induced liver damage [24], and NAFLD [21].

A defining characteristic of ALD is the presence of hepatic oxidative stress [41,42]. The main sources of ROS production in hepatocytes are the mitochondria and the ER, primarily through the action of cytochrome P450 enzymes [42]. Acetaldehyde, a highly toxic metabolite of ethanol, plays a significant role in oxidative stress and cellular damage. As previously mentioned, it reacts with proteins to form acetaldehyde–protein adducts, which alter protein structure and function, impairing enzymatic activity and disrupting cellular processes [30]. Additionally, acetaldehyde interacts with lipids in cellular membranes, leading to lipid peroxidation and the generation of reactive aldehyde products like malondialdehyde (MDA) and 4-hydroxynonenal (4-HNE) [43]. Lipid peroxidation results in extensive damage to cellular lipids, proteins, and DNA, disrupting membrane integrity and fluidity, which contributes to cellular dysfunction and apoptosis. Moreover, the accumulation of acetaldehyde within cells can impair mitochondrial function, resulting in decreased ATP production and increased generation of ROS [44]. This creates a feedback loop that further amplifies oxidative stress and mitochondrial dysfunction. In this study, the Lieber–DeCarli ethanol diet significantly increased the levels of MDA, AOPP, O_2_^−^, NO_2_, and PBO in the liver tissue (Figure 1a–e). The increase in oxidative/nitrosative stress markers is indicative of excessive ROS production induced by ethanol, which results in lipid peroxidation and oxidative damage to cellular proteins and DNA, ultimately leading to hepatocellular necrosis, apoptosis, inflammation, and fibrosis. On the other hand, in mice fed with the Lieber–DeCarli ethanol diet, there was a marked reduction in the antioxidant potential of hepatocytes, evidenced by decreased activity of SOD, lower levels of GSH, and a diminished GSH/GSSG ratio (Figure 2b,c,g). Moreover, our findings revealed an increase in GSSG level accompanied by a reduction in total GSH level, while CAT activity remained unchanged (Figure 2a,d,e). These results demonstrated an altered pro-oxidant/antioxidant balance in the livers of mice with ALD. Similarly, various studies have consistently shown the presence of oxidative stress in ALD, characterized by a substantial reduction in antioxidant defense mechanisms. These findings include decreased levels of SOD, CAT, GPx, GR, GST, GSH, and the GSH/GSSG ratio [45,46,47]. Additionally, the downregulation of nuclear factor erythroid 2-related factor 2 (NRF2), a key transcription factor for antioxidant response, highlights the impairment of cellular defense mechanisms against oxidative stress [48]. Moreover, a recent study has reported elevated lipid peroxidation, evidenced by increased levels of TBARS, MDA, LOOH, and protein carbonylation, further supporting the role of oxidative stress in the pathogenesis of ALD [47]. The findings from our study provide strong evidence that alpinetin exerts a hepatoprotective effect, as it significantly reduces ROS and enhances antioxidative capacity. Treatment with alpinetin resulted in a significant reduction in oxidative/nitrosative stress markers, including MDA, AOPP, O_2_^−^, NO_2_, and PAB (Figure 1a–e). On the other hand, antioxidant markers, such as SOD, total GSH, GPx, and GSH, were notably increased (Figure 2b–d,f). Additionally, the GSH/GSSG ratio was significantly elevated, further indicating an improvement in antioxidant defense (Figure 2g). These antioxidative effects of alpinetin may be attributed to its ability to scavenge free radicals and neutralize singlet oxygen, which protects the liver from oxidative stress and supports liver recovery. A recent study has demonstrated that alpinetin reduces the generation of ROS and enhances thiol-dependent antioxidant systems, such as glutathione and thioredoxin, through the activation of NRF2 [48,49]. One of the mechanisms underlying the hepatoprotective effect of alpinetin is its inhibitory action on CYP2E1, an enzyme involved in alcohol metabolism that leads to the formation of highly toxic acetaldehyde [23]. An elevated level of hepatic CYP2E1 is one of the most reliable markers of excessive ROS production [50]. By inhibiting CYP2E1, alpinetin reduces oxidative stress and prevents lipid peroxidation, resulting in decreased levels of MDA and other reactive aldehydes that contribute to cellular damage [23]. Consistent with these findings, several studies have reported reductions in MDA levels, AOPP, or nitrosative stress in different animal models of liver injury when treated with various plant extracts, such as betaine and curcumin [51,52,53,54].

Excessive oxidative stress impairs the ability of the ER to maintain redox homeostasis, resulting in ER stress and subsequent cellular dysfunction [8,10,55]. Our study examined the effect of alpinetin on ER stress parameters in the livers of mice with ALD. In mice subjected to the Lieber–DeCarli ethanol diet, we observed a significant increase in GADD153, GRP78, and ATF4 levels (Figure 4a–c). GADD153, also known as CHOP (C/EBP homologous protein), is a key indicator of ER stress and apoptosis. Elevated GADD153 levels reflect enhanced ER stress, a common feature in ALD due to the accumulation of misfolded proteins and oxidative stress. Similarly, GRP78 is a chaperone protein that helps in protein folding within the ER and is a marker of ER stress. Increased GRP78 levels indicate an adaptive response to ER stress. ATF4, another critical ER stress-related transcription factor, is involved in the unfolded protein response (UPR). The significant elevation of ATF4 reflects an upregulated UPR, which is typically activated under conditions of severe ER stress [8,56]. Our results align with recent studies highlighting the role of ER stress and the potential for natural compounds, such as curcumin and betaine, to alleviate it effectively [57,58,59]. In the present study, alpinetin supplementation resulted in a reduction in the protein expression of GADD153, GRP78, and ATF4, highlighting its potential as a therapeutic agent (Figure 4a–c). The exact mechanisms by which alpinetin stabilizes the ER and reduces stress are not fully understood. However, some proposed mechanisms include the downregulation of signaling pathways, such as the IRE-1/XBP-1 pathway [60].

Inflammation plays a central role in the progression of ALD, contributing to the pathogenesis and exacerbation of liver damage [31]. Chronic alcohol consumption leads to the activation of immune cells, particularly neutrophils, which release pro-inflammatory cytokines. These include TNF-α, interleukins (such as IL-1, IL-4, IL-6, IL-10, IL-12, IL-17, and IL-22), IFN-γ, C-reactive protein, TGF-β, and adiponectin [33]. Inflammatory cascade not only amplifies oxidative stress but also promotes hepatocellular injury, apoptosis, and fibrosis. As previously mentioned, significant neutrophil infiltration was found in the liver tissue of mice from the ethanol-fed group, with a histological score markedly higher than that observed in the livers of control animals (Figure 5B). The persistent inflammatory response, exacerbated by alcohol-induced gut permeability and endotoxemia, creates a vicious cycle of liver injury [32,33]. Our study demonstrated increased levels of inflammatory cytokines, specifically IFN-γ and IL-4, in the liver tissue of mice subjected to the Lieber–DeCarli ethanol diet (Figure 3a,b). The elevated cytokine levels observed are consistent with the established role of these cytokines in the inflammatory response associated with ALD [31]. IFN-γ is known to exacerbate inflammation and liver injury by promoting pro-inflammatory pathways, while IL-4, despite its role in anti-inflammatory responses, can contribute to fibrogenesis and further liver damage under chronic inflammatory conditions [61,62]. Alpinetin supplementation significantly reduced the liver concentrations of both IFN-γ and IL-4 in ethanol-fed mice (Figure 3a,b). These findings suggest that alpinetin has a potent anti-inflammatory effect, effectively mitigating the inflammatory response triggered by chronic alcohol exposure. Our study also assessed MPO activity, an enzyme released by neutrophils and a marker of inflammation [63]. Elevated MPO activity in the liver tissue of ethanol-fed mice confirms the presence of neutrophil infiltration and increased oxidative stress (Figure 3c). However, alpinetin treatment led to a significant reduction in MPO activity, indicating that it can attenuate inflammation and oxidative damage (Figure 3c). Alpinetin exerts its anti-inflammatory effects by modulating multiple signaling pathways, including inhibiting the NF-κB and MAPK pathways, activating the NRF2 pathway, and affecting the TLR and JAK-STAT pathways [24,64,65,66]. Additionally, a recent study has explored the relationship between alpinetin and methyl-CpG-binding protein 2 (MeCP2), revealing their combined role in modulating inflammation. The study indicates that MeCP2, an epigenetic regulator known for its ability to bind methylated DNA and modulate gene expression, promotes the anti-inflammatory effects of alpinetin through epigenetic modification crosstalk. Specifically, MeCP2 influences the transcription of pro-inflammatory genes, and the introduction of alpinetin amplifies this regulatory effect, further suppressing inflammation [67]. This suggests that the anti-inflammatory effects of alpinetin might not only be due to direct modulation of signaling pathways but also occur through modifications in gene expression.

## 4. Materials and Methods

### 4.1. Diet and Chemicals

The control diet and Lieber–DeCarli ethanol liquid diet were purchased from Bio-Serv, Canada, while ethanol was obtained from Sigma-Aldrich, Co., St. Louis, MO, USA.

### 4.2. Experimental Animals and Design

The experiment was performed on adult, wild-type C57BL/6 male mice weighing 17–20 g, raised at the Military Medical Academy in Belgrade, Serbia. During the experiment, the animals were kept under standard laboratory conditions (temperature 22 ± 2 °C, relative humidity 50 ± 10%, 12/12 h light/dark cycle, with lights turned on at 9.00 a.m.). The mice were acclimatized for 5 days with a control Lieber–DeCarli ethanol liquid diet (F1259SP, Bio-Serv, Flemington, NJ, USA) *ad libitum*. All experimental procedures were conducted according to the guidelines established in the Directive of the European Parliament and of the Council (2010/63/EU) and approved by The Ethical Committee of the University of Belgrade (No. 695/2).

All the animals (n = 23) were divided into the following groups: 1. control group (n = 6), 2. ethanol-treated group (E; n = 8), and 3. E group treated with alpinetin (E+Alp; n = 9). The E and E+Alp groups were fed with Lieber–DeCarli liquid ethanol diet (5% vol/vol ethanol) for 10 days, and on day 11, each mouse received a single dose of maltose dextrin (3585; Bio-Serv) and 36.5% (*vol*/*vol*) ethanol (5 g/kg) by oral gavage [16]. Alpinetin in a dose of 10 mg/kg was dissolved in distilled water and given to the mice via gavage on the last five days of the experimental period (E+Alp) [19], while control animals received the same amount of distilled water by oral gavage.

The animals of the control group were treated with a control liquid diet that had the same caloric value as the Lieber–DeCarli diet but without ethanol and with the substitution of maltose dextrin. On day 11, control animals received saline. The composition of the Lieber–DeCarli and the control liquid diet are presented in Table 3 All animals had free access to the appropriate diet during the experiment. Animals were sacrificed 9 h after oral gavage by cardiac puncture under ketamine anesthesia (100 mg/kg intraperitoneally (i.p.)), and liver samples were collected for morphological analysis of the tissue. Collected serum and liver tissue samples were stored at 20 °C and 80 °C, respectively, for analysis.

### 4.3. Preparation of the Blood and Tissue Samples

Blood samples were taken from the right side of the heart by cardiac puncture under ketamine anesthesia (100 mg/kg i.p.). Blood was collected in dry tubes, and serum was obtained by centrifugation at 1500× *g* for 10 min. Serum samples were stored at −80 °C until required for analysis. Liver samples for biochemical analysis were homogenized on ice in cold buffered 0.25 M sucrose medium (Serva, Feinbiochemica, Heidelberg, New York, NY, USA), 10 mM phosphate buffer (pH 7.0), and 1 mM ethylenediaminetetraacetic acid (EDTA, Sigma Chem. Co., St. Louis, MO, USA). The homogenates were centrifuged at 2000× *g* for 15 min at 4 °C. Coarse sediments were dissolved in a sucrose medium and centrifuged. The supernatants were transferred into the tubes and centrifuged at 3200× *g* for 30 min at 4 °C. Obtained sediments were dissolved in deionized water. After one hour of incubation, the samples were centrifuged at 3000× *g* for 15 min at 4 °C, and the supernatants were stored at −70 °C. Liver samples were taken for the determination of nitrosative and oxidative/antioxidative parameters and histopathology analysis.

### 4.4. Determination of Serum Activity of ALT, AST, γ-GT, and ALP

Biochemical evaluation of liver injury was performed by determination of the activity of serum alanine aminotransferase (ALT), aspartate aminotransferase (AST), gamma-glutamyl transferase (γ-GT), and ALP. Activities of these enzymes were measured spectrophotometrically with a photometer BTS-330 according to the manufacturer’s instructions using special kits containing 2-oxoglutarate (Sigma-Aldrich, St. Louis, MO, USA) for ALT and AST. γ-GT measurements were conducted using a commercially available kit (Olympus Diagnostics, Hamburg, Germany). The activity of ALP was measured using a kit containing 4-nitrophenyl phosphate (Sigma-Aldrich).

### 4.5. Determination of Oxidative/Nitrosative Stress Parameters and Antioxidative Markers in the Liver Tissue

The liver was harvested, weighed, and stored at −80 °C for later analysis. MDA (malondialdehyde), as a marker of lipid peroxidation, was measured in liver homogenates by using 4,6-dihydroxy-2-mercaptopyrimidine according to the method of Mihara and Uchiyama [68]. Advanced oxidation protein products (AOPPs), a marker of protein oxidative damage, were measured in an acidic condition in the presence of potassium iodide using the method of Selmeci et al. [69]. The concentration of nitrite (NO_2_^−^) in liver tissue was quantified using the Griess reagent method, following standard protocols [70]. The superoxide anion radical (O_2_^−^) levels were assessed by its reaction with nitro blue tetrazolium in TRIS buffer, and measurement was performed at 530 nm [71]. The pro-oxidant–antioxidant balance (PAB) in the liver tissue was measured using 3,30,5,50-tetramethylbenzidine as a chromogen [72]. The activity of the myeloperoxidase enzyme was measured using the o-dianisidine-H2O2 method, as described by Kothari et al., with minor modifications [73]. The activity of antioxidant enzymes, superoxide dismutase (SOD), catalase (CAT), and glutathione peroxidase (GPx) was determined in liver homogenates using the spectrophotometric method, as previously described [74,75]. The SOD converts superoxide radicals into hydrogen peroxide and molecular oxygen, while CAT and peroxidases convert H_2_O_2_ into water. CAT activity was calculated using the molar extinction coefficient and normalized to the concentration of protein. One SOD unit was defined as the amount of enzyme required to inhibit 50% of the adrenaline and normalized to the protein concentration. The total glutathione (GSH) in the liver tissue was quantified following the method by Sies and Akerboom [76]. The method for determining reduced GSH involved the reduction reaction of GSH disulfide to GSH, using NADPH as the reducing cofactor [77]. The content of oxidized GSH was obtained from the difference between total and reduced GSH. The redox state of the liver was determined spectrophotometrically using an UltroSpect 3300 Pro UV/Visible spectrophotometer (Amersham Biosciences Corp., Piscataway, NJ, USA).

### 4.6. Determination of Cytokines in the Liver Tissue

For the determination of cytokines (INF-γ and IL-4) in liver tissue, samples were homogenized in 10 volumes of PBS. After centrifugation (10 min at 12,000× *g*, 4 °C), the supernatants were carefully collected and diluted to 1/40,000 in PBS [78]. The concentration of cytokines (IFN-γ and IL-4) was determined using ELISA kits from BD Bioscience (San Diego, CA, USA) according to the manufacturer’s instructions.

### 4.7. Examination of ER Stress Parameters in the Liver

Liver tissues were harvested, weighed, and stored at −70 °C for later analysis. Previously frozen liver tissue was homogenized with phosphate buffer. Equal amounts of protein (30 μg) were run on polyacrylamide gels and transferred to nitrocellulose membranes. The membranes were blocked with 3% milk (Serva Electrophoresis GmbH, Heidelberg, Germany) for 1 h at room temperature and probed with primary antibodies directed against GADD153 (Santa Cruz Biotechnologies, Dallas, TX, USA), GRP78 (Santa Cruz Biotechnologies, Dallas, TX, USA), ATF4 (Santa Cruz Biotechnologies, Dallas, TX, USA), and β-actin (Santa Cruz Biotechnologies, Dallas, TX, USA). The GADD153, GRP78, and ATF4 protein levels were estimated by densitometric scanning of the blots using the Image Master Total Lab (Version 7, GE Healthcare, Amersham, UK) software tool and normalized to that of β-actin.

### 4.8. Histopathology of the Liver

After collecting the liver samples, they were fixed for 12–24 h in 10% buffered formalin and then rinsed with distilled water. Over the next 24 h, the samples were dehydrated in alcohols of increasing concentration (from 70% to absolute alcohol). After dehydration, the samples were cleared in xylene and then embedded in paraffin blocks. The paraffin blocks were sectioned using a standard microtome into slices 3–5 μm thick. The sections were then stained with hematoxylin–eosin and Masson Trichrome. All factors of steatohepatitis (steatosis, hepatocyte injury (ballooning), and inflammation) were evaluated using the SALVE grading and staging system [79].

### 4.9. Statistical Analysis

The data were analyzed by the statistical software package STATISTICA 12, StatSoft Inc., (Tulsa, OK, USA). All results are presented as mean ± SD. The normality of the dataset was verified using the Kolmogorov–Smirnov test. To assess group differences, a one-way analysis of variance (ANOVA) followed by Tukey’s post hoc test was performed. Statistical significance was defined at *p* < 0.05.

## 5. Conclusions

This study demonstrated that alpinetin offers significant hepatoprotective effects in a model of ALD induced by the Lieber–DeCarli ethanol diet. Our findings are consistent with previous studies that have reported the hepatoprotective effects of various natural compounds in ALD models. Similar to other flavonoids, alpinetin appears to ameliorate liver injury through its antioxidant and anti-inflammatory actions. To the best of our knowledge, this is the first study to investigate the potential effects of alpinetin in the ALD model and, more specifically, the first to explore its influence on ER stress.

## Figures and Tables

**Table 1:** The effects of alpinetin on body weight, liver weight, liver index, and body weight loss.

Groups	Body Weight (BW) (g)	Liver Weight (LW) (g)	Liver Index (%) LW/BW %	Body Weight Loss (%)
C	21.93 ± 1.56	1.21 ± 0.04	5.53 ± 0.41	−19.10 ± 9.54
E	16.31 ± 1.01 ^###^	1.34 ± 0.17 ^###^	8.21 ± 1.09 ^###^	13.21 ± 3.15 ^###^
E+Alp	16.9 ± 0.44 ^###^	1.17 ± 0.08 *	6.95 ± 0.5 ^#,^**	5.47 ± 4.44 ^###,^*

The values are represented by mean ± SD (n = 7). ^#^ *p* < 0.05, ^###^ *p* < 0.001 vs. C; * *p* < 0.05, ** *p* < 0.01 vs. E. Abbreviations: C—control group; E—ethanol group; E+Alp—ethanol + alpinetin group.

**Table 2:** The effects of alpinetin on serum ALT, AST, and γ-GT activity.

Groups	ALT (U/I)	AST (U/I)	AST/ALT Ratio	γ-GT (U/I)
C	33.78 ± 6.96	86.32 ± 13.34	2.6 ± 0.43	6.38 ± 1.74
E	175.0 ± 42.5 ^###^	381.1 ± 117.2 ^###^	3.3 ± 0.43 ^###^	20.96 ± 1.59 ^###^
E+Alp	59.04 ± 9.9 ***	193.1 ± 42.1 ***	3.17 ± 0.67 ^###,^**	16.93 ± 0.75 ^###,^***

The values are represented by mean ± SD (n = 7). ^###^ *p* < 0.001 vs. C; *** *p* < 0.001, ** *p* < 0.01 vs. E. For abbreviations, see Table 2. ALT—alanine aminotransferase; AST—aspartate aminotransferase; γ-GT—gamma-glutamyl transpeptidase.

**Table 3:** The composition of the Lieber–DeCarli alcoholic diet and the control diet.

Content	Lieber–DeCarli Diet	Control Diet
Dry mix (g)	133	225
Maltose dextrin (g)	20.3	89.9
Water (mL)	910	860
95% ethanol (mL)	52.6	0

The diets had equal caloric values, with maltose dextrin substituting for ethanol in the control diet. Alpinetin, sourced from Sigma-Aldrich Chemical Co. (St. Louis, MO, USA), was dissolved in distilled water. The prepared solution was administered to C57BL/6 mice at a dosage of 10 mg/kg (5 mL/kg).

## Data Availability

The dataset is available on request from the authors.

## Supplementary Materials

The following supporting information can be downloaded at: https://www.mdpi.com/article/10.3390/ijms26010086/s1.
