# Supplementary material for: Alpinetin Exhibits Antioxidant and Anti-Inflammatory Effects in C57BL/6 Mice with Alcoholic Liver Disease Induced by the Lieber–DeCarli Ethanol Liquid Diet
Source: Int J Mol Sci. 2024 Dec 26;26(1):86. doi: 10.3390/ijms26010086 (PMC11720451; doi:10.3390/ijms26010086)
Supplement: Supplementary file 1 [file ijms-26-00086-s001.zip › Figure S1.pdf]

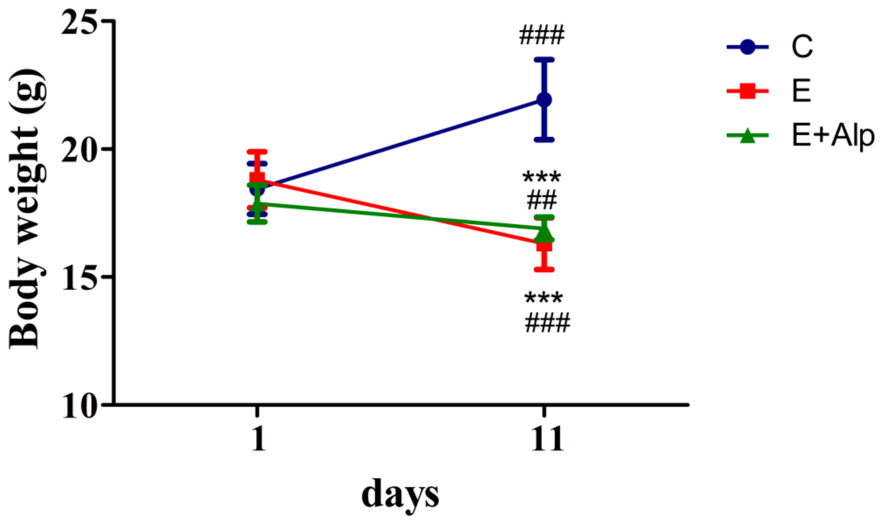


**Figure S1. Body weight of mice in the C control group, the E ethanol group and the E+Alp ethanol+alpinetin group at onset and end of the experimental period.** Alpinetin at a dose of 10 mg/kg was administered by gavage to the mice on the last five days of the experimental period (E+Alp), while the control animals received the same amount of distilled water by oral gavage. The values are represented by mean ± SD (n=7). ## $p < 0.01$; ### $p < 0.001$; vs. values at onset from the same group; *** $p < 0.001$; vs. C.
